# Behavior of Alkali-Activated Fly Ash through Underwater Placement

**DOI:** 10.3390/ma14226865

**Published:** 2021-11-14

**Authors:** Zarina Yahya, Mohd Mustafa Al Bakri Abdullah, Long-yuan Li, Dumitru Doru Burduhos Nergis, Muhammad Aiman Asyraf Zainal Hakimi, Andrei Victor Sandu, Petrica Vizureanu, Rafiza Abd Razak

**Affiliations:** 1 Centre of Excellence Geopolymer and Green Technology (CEGeoGTech), Universiti Malaysia Perlis (UniMAP), Perlis 01000, Malaysia; mustafa_albakri@unimap.edu.my (M.M.A.B.A.); rafizarazak@unimap.edu.my (R.A.R.); 2 Faculty of Civil Engineering Technology, Universiti Malaysia Perlis (UniMAP), Perlis 01000, Malaysia; aimanzasyraf@gmail.com; 3 Faculty of Chemical Engineering Technology, Universiti Malaysia Perlis (UniMAP), Perlis 01000, Malaysia; 4 School of Marine Science and Engineering, University of Plymouth, Plymouth PL4 8AA, UK; long-yuan.li@plymouth.ac.uk; 5 Faculty of Materials Science and Engineering, Gheorghe Asachi Technical University of Iaşi, Boulevard D. Mangeron, No. 51, 700050 Iasi, Romania; dumitru-doru.burduhos-nergis@academic.tuiasi.ro; 6 Romanian Inventors Forum, Str. P. Movila 3, 700089 Iasi, Romania

**Keywords:** alkali-activated, underwater placement, class C fly ash, seawater, fresh water

## Abstract

Underwater concrete is a cohesive self-consolidated concrete used for concreting underwater structures such as bridge piers. Conventional concrete used anti-washout admixture (AWA) to form a high-viscosity underwater concrete to minimise the dispersion of concrete material into the surrounding water. The reduction of quality for conventional concrete is mainly due to the washing out of cement and fine particles upon casting in the water. This research focused on the detailed investigations into the setting time, washout effect, compressive strength, and chemical composition analysis of alkali-activated fly ash (AAFA) paste through underwater placement in seawater and freshwater. Class C fly ash as source materials, sodium silicate, and sodium hydroxide solution as alkaline activator were used for this study. Specimens produced through underwater placement in seawater showed impressive performance with strength 71.10 MPa on 28 days. According to the Standard of the Japan Society of Civil Engineers (JSCE), the strength of specimens for underwater placement must not be lower than 80% of the specimen’s strength prepared in dry conditions. As result, the AAFA specimens only showed 12.11% reduction in strength compared to the specimen prepared in dry conditions, thus proving that AAFA paste has high potential to be applied in seawater and freshwater applications.

## 1. Introduction

The construction of structures involving concrete underwater placement usually require additional considerations due to its unique circumstances. Typically, the effective placement of conventional concrete mixture underwater depends on two main factors: the mix design of concrete itself and placement method during concreting [1,2]. For the mix design of concrete, additions of anti-washout admixtures (AWA) and viscosity-modifying admixtures (VMAs) are necessary for conventional concrete to minimise the washout effect and the ability to self-consolidate during the underwater placement [3,4,5]. Concrete resistance against washout can also be improved using mineral admixture with high fineness. The most used mineral admixture includes silica fume, ground granulated blast furnace slag (GGBS) and fly ash (FA) [2,6]. Heniegal [7] confirmed that the inclusion of fly ash and silica fume with the addition of limestone or bentonite powder improved the flowing properties of conventional concrete and minimised the washout effect. 

Past researchers had also investigated the use of seawater as replacement for water in ordinary Portland cement (OPC) concrete. The justification for using seawater in concrete is for offshore structures where it involves underwater concreting. According to Wang et al. [8] it is possible to mix seawater with cement where the early strength increased due to the existence of Cl^−^ and Na^+^ ions. Specimens with low water/cement (w/c) ratio showed more significant early strength development. For 28 days strength, it also showed an increment about 10% compared to specimen produced with fresh water but it induced corrosion on the rebars [9].

Meanwhile for the placement method, the concreting process can be made using Tremie pipe for mass concrete. This method required steel pipe with a hopper attached to the upper end and injectable plug on the bottom of pipe. The pipe is immersed in water and when the pipe is full of concrete, its bottom is opened for the concreting process. Using this technique, a hydro crane is required to lift the Tremie pipe after finishing concrete placement and the bottom of the pipe need to be kept in the fresh concrete during the process to avoid washout effect [2]. For a small concrete placement underwater, the skip and toggle bag methods are most suitable [1]. The concrete is filled up into different sized buckets, where the top covers are sealed to prevent water infiltration during the lowering process of the concrete placement. The bottom door of the bucket is slowly opened during concreting to allow free flowing of the concrete. 

Moreover, for underwater concrete mostly refer to the Standard by Japan Society of Civil Engineers (JSCE). This standard stated that the w/c ratio should be in range 0.50 to 0.55 when placing reinforced concrete in seawater and fresh water [10]. The w/c ratio can be increased up to 0.60 and 0.65 when concreting for non-reinforced concrete. For the strength of hardened concrete through underwater placement, JSCE standards required the compressive strength of specimens attain a minimum of 80% strength with respect to specimen cast in dry conditions [2,10]. 

The manufacturing of ordinary Portland cement (OPC) consumes a lot of natural resources, is energy intensive and contributed to carbon dioxide (CO_2_) emission to the atmosphere [11]. It was estimated that 7% of CO_2_ emission comes from the OPC industry [12], which is about 1.35 billion tons per annum. This is a serious environmental concern, and research endeavours involve finding a suitable alternative binder to replace OPC in concrete. The literature [13] refers to the work of Davidovits that found geopolymer in 1978 which also known as amorphous alkali aluminosilicate, and are sometimes referred to as inorganic polymers, geocements, or alkali-activated cements. This new alternative binder is produced by activating source materials with alkaline activators, and its classification is dictated by the content of silica, aluminium, and calcium. If the source materials are made up of mainly silica and aluminium (Class F fly ash, metakaolin, or some natural pozzolan), its final product is the sodium aluminosilicate hydrate (N-A-S-H) backbone of the geopolymer [14]. If the source materials are made up of calcium, aluminium, and silica (Class C fly ash and slag), then the main product after hardening is calcium silicate hydrate (C-S-H) or calcium alumino silicate hydrate (C-A-S-H), which also can be described as alkali-activated materials (AAM) [15]. 

Fly ash is an industrial waste material that is ubiquitous due to the increasing demand for energy, which is met by increasing coal-fired power plant’s usage. The world coal production is expected to rise between 2006 and 2030 by almost 60%, with volumes output to 7011 Mtce by 2030 [16,17,18,19]. The management of fly ash disposal is always concerned by environmentalist since only 20–30% of the generated fly ash is reused whereas the rest was disposed either in landfills or ponds [17,18]. Therefore, the use of fly ash as aluminosilicates sources in AAM production is a waste-to-health approach that could also mitigate environmental concerns. 

The parameters that influence the properties of AAM have been intensively investigated [19,20,21,22,23,24,25], and AAM are known to be resistant against aggressive ions, freeze-thaw resistance, have high early and long-term strength, and excellent fire resistance [13,26,27,28,29,30]. The main issue of using conventional concrete for underwater structure is its resistance to washout. Concrete resistance to washout depends on the content of fine fraction in the binder, water cement ratio, and cement content. Concrete resistance to washout depends on the content of fine fractions in the binder, water cement ratio, and cement. Previous studies are mainly focused on underwater concrete placement using OPC as a binder and addition of special admixture for construction offshore structures such as bridge piers, but studies involving the application of AAM for underwater concreting remain scarce. The current study investigated the performance of alkali-activated fly ash (AAFA) paste through underwater placement in seawater and freshwater (river water and lake water). The compressive strength, changes in pH, X-ray fluorescence (XRF) and Field Emission Scanning Electron Microscope coupled with Energy Dispersive X-ray spectroscopy (FESEM-EDS) are analysed, respectively.

## 2. Materials and Methods

### 2.1. Materials

In this study, fly ash was used as source materials for AAM which is supplied by Cement Industries of Malaysia Berhad (CIMA), Perlis, Malaysia. Noted that the major elements in fly ash are silica (SiO2), alumina (Al_2_O_3_), ferum (Fe_2_O_3_) and calcium (CaO). According to the American Society for Testing Materials (ASTM C618), the ash containing more than 70 wt.% of SiO_2_, Al_2_O_3_, Fe_2_O_3_, and low CaO is considered to be Class F; while that with total of SiO_2_, Al_2_O_3_, and Fe_2_O_3_ ranging within 50–70 wt.% defined Class C. Due to the relatively high calcium content (22.30%), the fly ash used in this experiment is classified as Class C according to the ASTM C618 [31]. 

Waterglass or sodium silicate solution was supplied by South Pacific Chemical Industries Sdn. Bhd. (SPCI), Malaysia. The waterglass consists of 30.1% SiO_2_, 9.4% Na_2_O and 60.5% H_2_O (modulus SiO_2_/Na_2_O = 3.2). Its specific gravity and viscosity are 1.4 g/cm^3^ and 0.4 Pas, respectively.

Sodium hydroxide (NaOH) powder brand Formosoda-P from Taipei, Taiwan with 99% purity was used. The desired concentration of NaOH solution was prepared 24 h before experiments by diluting NaOH powder with distilled water. The activator solution was prepared by mixing waterglass and NaOH solution at a ratio of 2.5.

### 2.2. Collection of Water Samples

In this study, the seawater, river water, and lake water samples are collected around Perlis, Malaysia. The water collected was left in the laboratory to allow the impurities to precipitate at the container’s bottom. Later, the water was transferred to a plastic tank via infiltration and the pH value for each type of water was recorded.

### 2.3. Specimens Preparation

**Figure 1:**
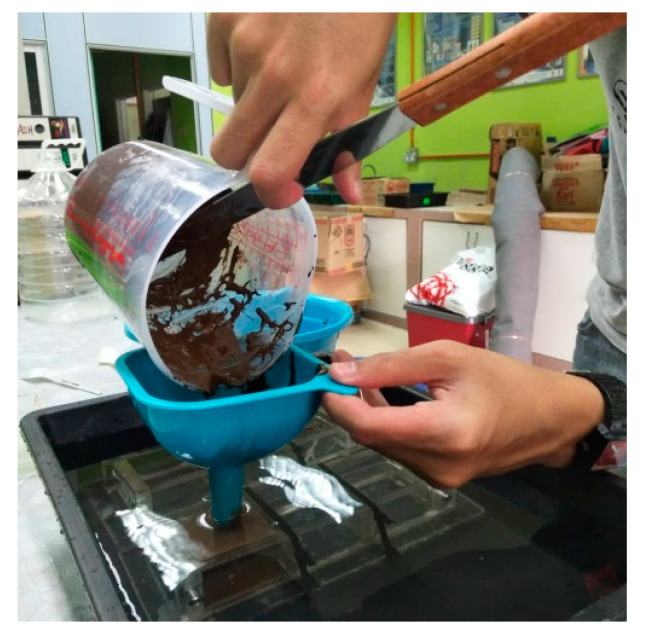
AAFA paste poured into mould in seawater.

The concentration of NaOH solution is fixed at 12 M [32], the ratio of waterglass-to-NaOH and ratio fly ash-to-alkaline activator fixed at 2.0 and 2.5 respectively [22]. The details of mix design are summarized in Table 1. The fly ash and alkaline activator were mixed and stirred for 5 min using a mechanical mixer. Then the fresh AAFA paste poured into the 50 mm × 50 mm × 50 mm [33] moulds that already placed in a container with seawater, river water, and lake water as shown in Figure 1. The AAFA specimens were left in the container for 3, 7, and 28 days, respectively. The pH level and temperature of the water before and after the placement of AAFA paste were recorded. For the control specimens, the AAFA paste was prepared in dry conditions (without underwater placement).

### 2.4. Testing and Analysis Methods

The setting time of AAFA paste through underwater placement was measured using the Vicat test [33]. The test was conducted at room temperature using the Vicat apparatus, where the mould was placed beneath the water level. The initial setting and final setting time of the AAFA paste were recorded. 

The specimen’s compressive strength was determined based on ASTM C109 [34] using Instron 5582 Mechanical Tester (Instron, Massachusetts, United States America). A minimum of three specimens was tested for each mix design, and the average results recorded. Total of all 108 specimens were produced for this testing. The AAFA were tested on 3rd days, 7th days, and 28th days for both the control specimens and specimens cast underwater.

The chemical composition of the AAFA paste after going through underwater placement and dry condition is determined using X-ray fluorescence (XRF). XRF was conducted using the PA Nanalytic PW 4030, MiniPAL 4 (Malvern Panalytical, Malvern, United Kingdom) X-ray fluorescence spectrometer. After 28 days placement in various water types, the specimens were crushed to a powder form for analyses.

A JSM-7001F (JEOL, Tokyo, Japan) model of Field Emission Scanning Electron equipped with energy dispersive spectroscopy (EDS) was used to image the AAFA’s morphology and determine its elemental composition after underwater placement. The specimens were cut into small pieces and coated with carbon using Auto Fine Coater (JEOL, Tokyo, Japan). The images were observed with accelerating voltage of 15 kV for all specimens.

## 3. Results and Discussion

### 3.1. pH Value and Temperature of Water

**Figure 2:**
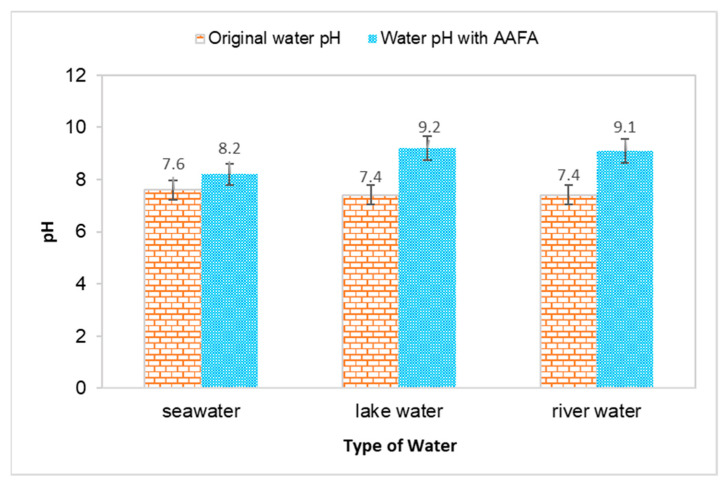
Effect on pH value for different type of water due to placement of the AAFA paste.

**Figure 3:**
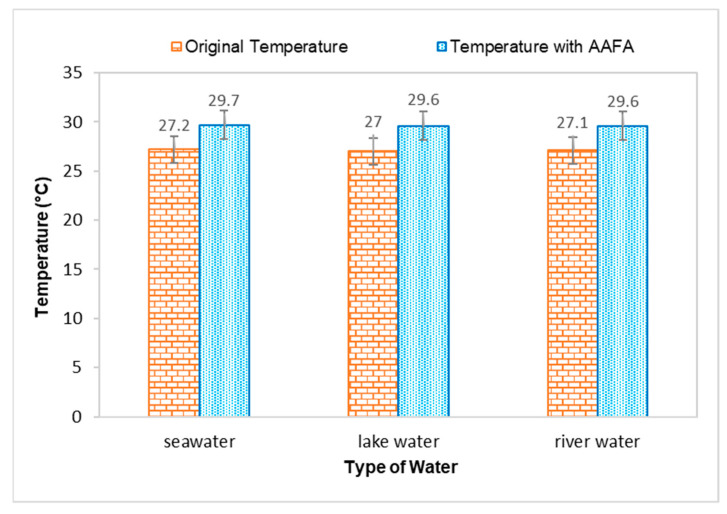
Effect on temperature when casting the AAFA in water.

The water’s pH value and temperature before and after underwater placement of the AAFA are illustrated in Figure 2 and Figure 3. The seawater’s original pH is 7.5, while the river water and lake water pH value are 7.4. All types of water recorded an increment in value when the AAFA paste was placed into the tanks. The seawater’s pH value recorded the lowest increment of 0.6, while the highest increment was 1.8 from the lake water. From the underwater placement of AAFA paste, there is washout effect with the increment in the surrounding water’s pH value. However, visible changes in the pH are evident, probably due to the smaller tank (275 mm length × 160 mm width × 160 mm depth) used during underwater placement of the AAFA specimens. 

The temperature for all types of water increased when the AAFA paste was placed into the tanks, proving that the AAFA reaction is exothermic, as heat is released during the hardening process, which increased the temperature of the water. Temperature increment in the seawater and river water was 2.5 °C, while lake water had a temperature increment of 2.6 °C. Previous researchers reported that the reaction between the source materials and alkaline activator is an exothermic reaction when the AAFA is cured at high temperature [33,34,35,36,37]. However, it was observed in this study that even though the AAFA paste was placed in water, heat release can still be detected.

### 3.2. Setting Time

The setting time of cement or binder occurred when it loses its plasticity and slowly formed into hard rock type material. The initial setting time can be defined as the time taken by the paste to start stiffening; whereas the final setting time is when the paste begins to harden and able to carry some loads. The initial and final setting times for the AAFA paste through underwater placement in various water types are shown in Table 2. For the initial setting time, the AAFA specimens placed in seawater were recorded at the fastest time of 26 min, while the river water specimens recorded with the longest time of 30 min. For final setting time, the specimens in seawater and lake water recorded the same value of 35 min and for river water it recorded 37 min. The final setting time of Class C fly ash in dry condition (room temperature) is usually between 1–2 h, depending on the content of calcium (CaO) [38]. For the current study, the initial and final setting time of the AAFA specimens casted in dry condition reported 31 min and 40 min, respectively. This finding in agreement with past research where it was found that Class C fly ash recorded initial and final setting times of 32.15 min and 60.00 min for specimens casted in dry condition [39]. Source materials rich in Ca content have quick setting time in dry condition due to the higher dissolution rate of Ca^2+^ compared to Si^4+^ and Al^3+^. The reaction product of the source materials rich in calcium is expected to form of Ca-rich phases that will develop the fundamental skeleton of the AAFA network. According to previous research, reaction products such as calcium silicate hydrate (C-S-H), calcium aluminate silicate hydrate (C-A-S-H) and sodium calcium aluminate silicate hydrate (N, C-A-S-H) are expected to be present in Ca-rich phase [40]. 

The quick setting time for underwater placement of the AAFA in seawater is due to Cl^−^ ions which react with cations in the AAFA paste such as Na^+^ and Ca^2+^. The formation of calcium chloride (CaCl_2_) is widely known as an accelerator for early strength development as well as a minimiser for the setting time [41]. Additionally, during underwater placement of the AAFA specimens, the existence of water helps to improve the properties of AAFA. According to Duxson et al. [42], water helps accelerate Si and Al dissolution process from the source materials by providing discontinuous gel nanopores to the paste, hence improved its performance. For practical application, it is suggested to use the retarding admixture to control the setting time, as it can delay the setting time and keep the AAFA concrete workable throughout the placing process.

### 3.3. Compressive Strength

**Figure 4:**
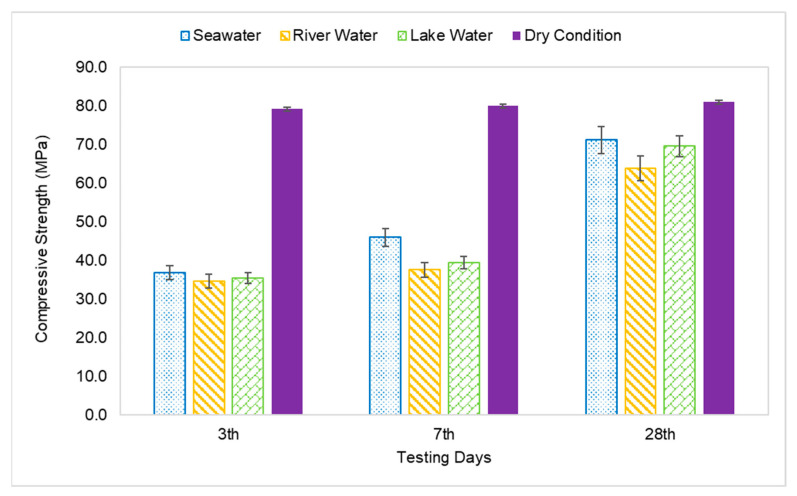
Compressive strength of AAFA paste cast in water.

The compressive strength of the AAFA specimens through underwater placement was evaluated on 3rd, 7th, and 28th days. All specimens displayed increment in strength with respect to aging days as per Figure 4. The AAFA specimens cast in seawater displayed the highest compressive strength for all the aging days; for example, on 3rd days, the AAFA specimens reported a strength of 36.8 MPa. Meanwhile, the lowest compressive strength was found in the specimens cast in river water with strength 34.6 MPa on 3rd days. For control specimens (dry condition), the compressive strength on 3rd days is 79.1 MPa. The specimens cast in seawater recorded a decrease in their compressive strength by 54% compared to the specimens cast in dry condition for 3rd days of testing.

The compressive strength of the AAFA specimens cast in seawater was reported to be 46.0MPa on the 7th days, whereas its dry counterpart has a reported strength of 79.9 MPa. This translates to a 20% strength increment in the AAFA specimens cast in seawater from day 3 to day 7. However, the strength increment from day 7 to day 28 was even higher, which is 55%. In contrast, the 28 day’s strength of the specimens cast in dry condition was found to be 80.9 MPa, indicating that it has a lower strength increment. According to Kumar et al. [43], the increment of strength with respect to time can be attributed to calcium silicate hydrate (C-S-H) formation. Further discussion about the reaction product will be provided in Section 3.4. 

The AAFA cast in dry condition exhibited almost complete strength development within 3 days, as the strength increment from 3rd days to 28th days testing was only 2%. For the AAFA cast in water, the strength slowly increased from 3rd days to 28th days for all water types. In the case of the 28-day strength, the AAFA specimens cast in seawater recorded a strength decrease by 12% relative to the specimen cast in dry condition, implying that the AAFA can be used for constructing a structure in water due to its impressive strength. Normally, conventional concrete (ordinary Portland cement) requires anti-washout admixture (AWA) and high range water reducer admixture (HRWR) before it can be used as binder materials in construction, especially for underwater structures. However, using AAFA only requires materials rich in silica and alumina, as well as alkaline activator. Additionally, the raw materials used in this case (silica and alumina sources) are waste materials, which falls in line with green technology promotion. 

### 3.4. Chemical Composition Analysis 

The chemical composition of control and AAFA specimens placed in different types of water is presented in Table 3. All the AAFA paste showed an increment in SiO_2_ content due to the reaction of the fly ash with waterglass (Na_2_SiO_3_). Meanwhile the content of Al_2_O_3_ showed a reduction in AAFA paste relative to the raw fly ash. This is due to the participation of Al_2_O_3_ in setting time of the AAFA via acceleration of the condensation of the AAFA product formation [44,45]. The content of Fe2O_3_ also increased in the AAFA paste, especially those cast in dry condition, hence contributing to the maximum compressive strength. It was suggested that Fe^3+^ contributed to the formation of AAFA network due to the similar charge and ionic radius with Al^3+^ [46,47]. However, the increment of most chemical composition between different specimens is almost similar, which is related to the compressive strength. The compressive strength of the AAFA depends on a few factors such as the formation of reaction products, distribution of Si-Al ratio, calcium content, and the surface reaction between the unreacted Si-Al particles [48,49]. 

The molar ratio of Si/Al, Ca/Si, and Fe/Si of raw fly ash and the AAFA paste was calculated based on the result from XRF. For the ratio of Si/Al, the compressive strength increased when the Si/Al ratio increased due to the formation of Si-O-Si bonds. The maximum ratio of Si/Al is contributed by the AAFA specimens cast in dry condition. Nevertheless, the ratios between the specimens do not differ much. 

The Ca/Si ratio for the source materials rich in Ca content is also linked with the compressive strength of the AAFA. The Ca/Si is responsible for the formation of C-S-H, and according to Timakul et al. [50], the compressive strength increased alongside the Ca/Si ratio. However, in this study, the ratio of Ca/Si is almost similar (~0.64–0.63) between the AAFA specimens cast in dry condition and seawater, which resulted in less of a difference in terms of compressive strength. For the AAFA cast in river water and lake water, the ratio of Ca/Si is similar (Ca/Si~0.62). Additionally, the formation of C-S-H, as AAFA reaction product and/or as OPC hydration product is entirely different. For the formation of C-S-H as hydration of OPC, the ratio of Ca/Si is in the range of 1.2 to 2.3, which is much higher relative to the AAFA [51,52]. The ratio of Fe/Si also plays essential role in forming the reaction product of the AAFA. The specimens’ compressive strength increased when the ratio of Fe/Si increased due to the formation of ferro-sialate-siloxo and/or ferro-sialate-disiloxo poly. The XRF result indicated that iron oxide is involved in the forming of the AAFA network and contributed to the AAFA’s strength. 

### 3.5. Microstructure and Elemental Composition of Reaction Products

**Figure 5:**
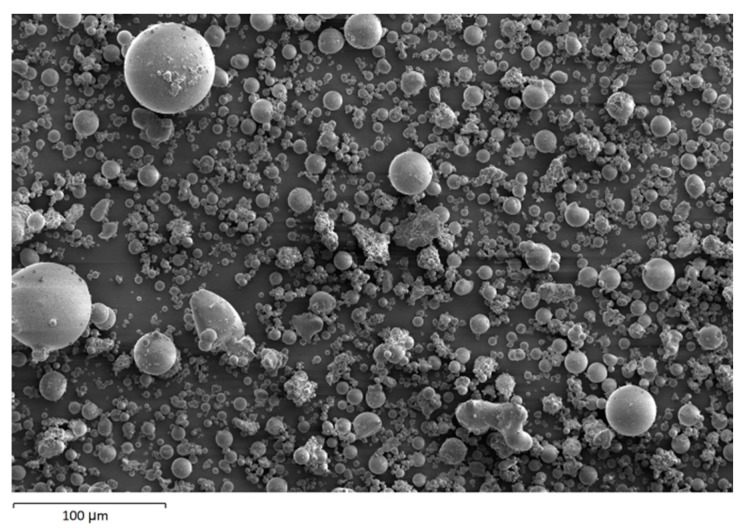
Morphology of fly ash.

**Figure 6:**
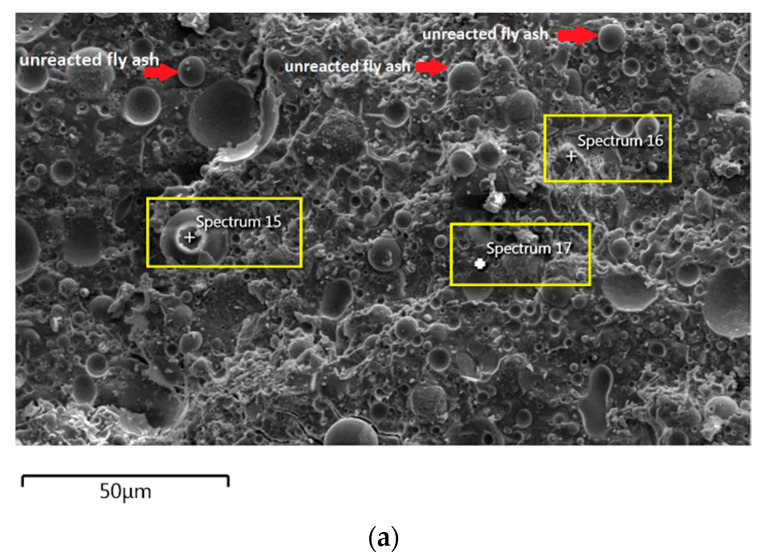

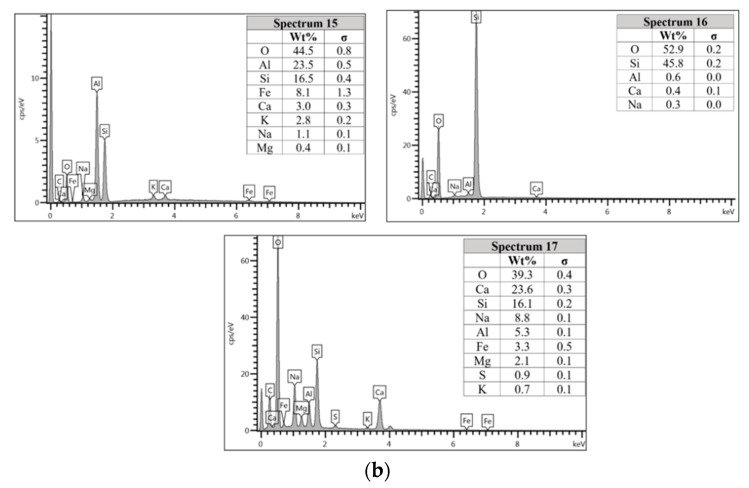
(**a**) Morphology of AAFA specimens cast in dry condition. (**b**) EDS for AAFA specimens cast in dry condition.

**Figure 7:**
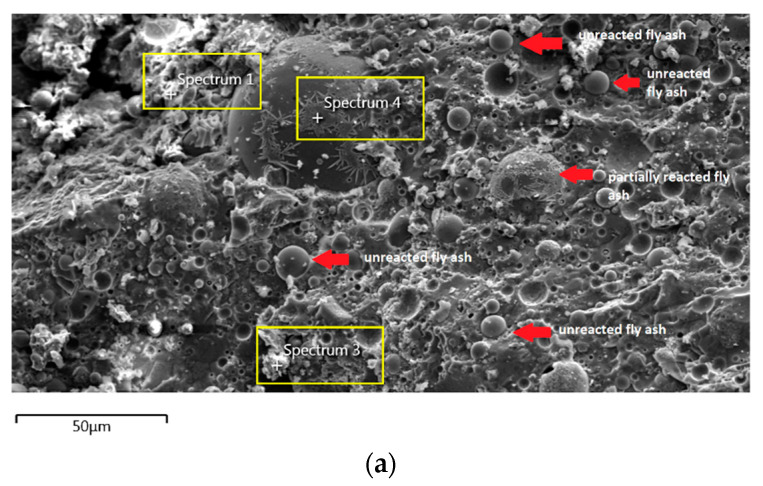

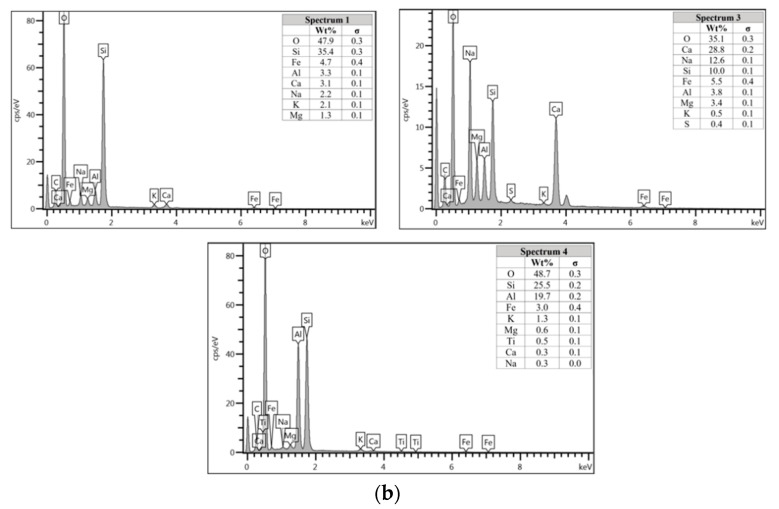
(**a**) Morphology of AAFA specimens cast in seawater. (**b**) EDS for AAFA specimens cast in seawater.

**Figure 8:**
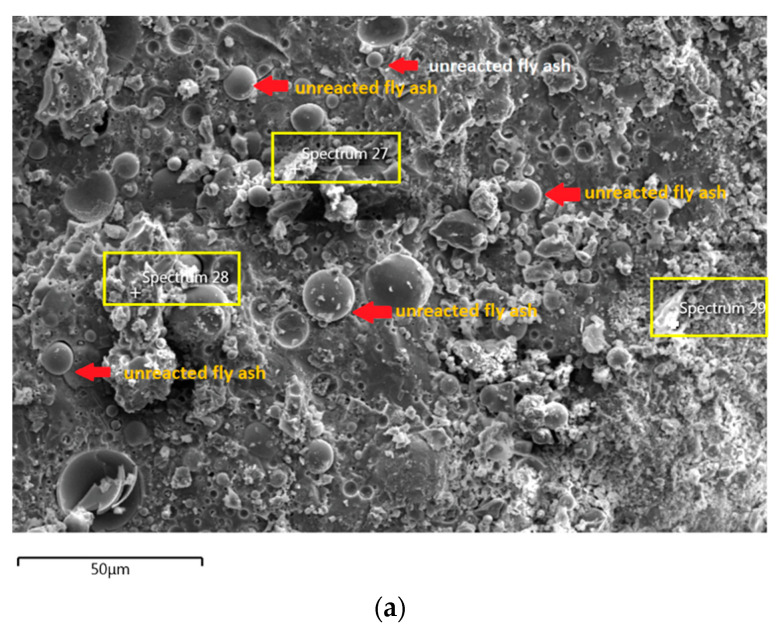

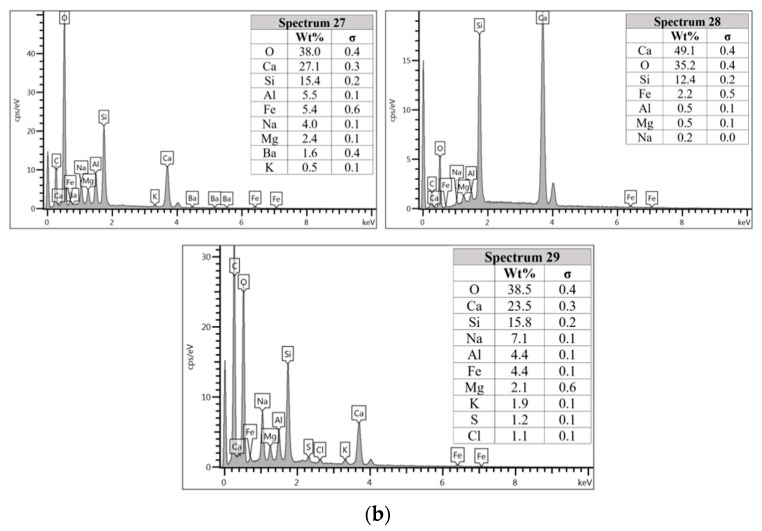
(**a**) Morphology of AAFA specimens cast in river water. (**b**) EDS for AAFA cast in river water.

**Figure 9:**
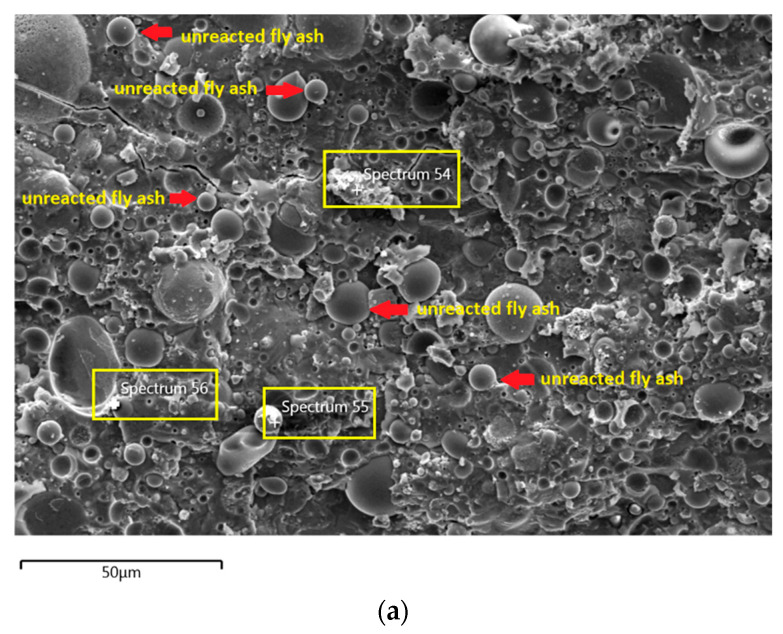

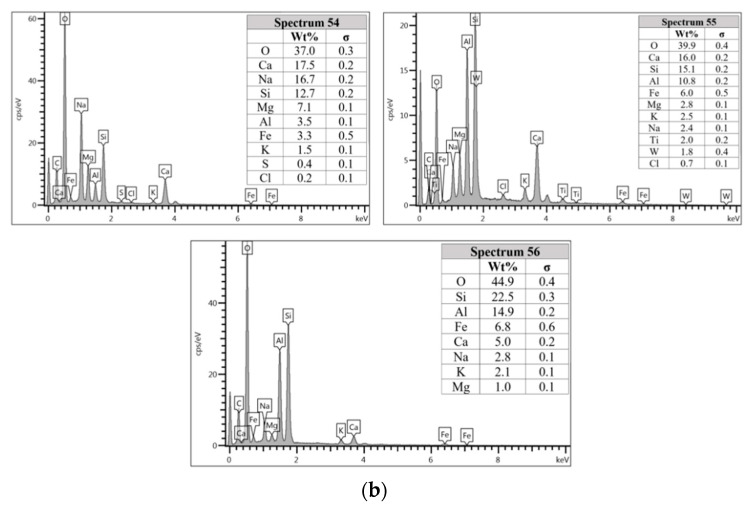
(**a**) Morphology of AAFA specimens cast in lake water. (**b**) EDS for AAFA specimens cast in lake water.

The morphology image of fly ash shown spheres particles shapes with smooth surface and various sizes of particles as in Figure 5. Figure 6, Figure 7, Figure 8 and Figure 9 show the microstructure and EDS of the specimens at three selected spots (represented by the spectrum numbers) in the matrix. Elements such as Si, Na, Fe, Al, Ca, and O were identified in the AAFA matrix for each specimen. The selected spot for each specimen is often different, which means that the EDS elemental composition is incomparable between each specimen. 

For the AAFA specimens casted in the dry condition as in Figure 6a, the present of unreacted fly ash still detected on the specimen. For the elemental composition of spectrum 15 is occupied by Si, Al, and Fe, with Ca and Na less than 5 wt.%. Referring to the FESEM images, spectrum 15 showed the particle shapes of fly ash. It can therefore be surmised that the unreacted fly ash contributed to the strength increment with respect to the aging period due to the complex reaction between the surfaces of the particles via bonding strength [53,54,55,56,57]. Meanwhile, the elemental composition in spectrum 16 majorly consists of Si, but for spectrum 17 is dominated by Si, Ca, Na, and Al as in Figure 6b. It can be hypothesised that these elemental compositions represent the reaction product of C-A-S-H and C-S-H due to the high content of Ca in the source material (fly ash).

Figure 7a shows the specimen cast in seawater where unreacted and partially reacted fly ash were detected. Through EDS analysis spectrum 1 is dominated mostly by Si with Na, Al, Ca, and Fe less than 5 wt.%. Spectrum 3 is dominated by Ca, Na, and Si, which indicate the formation of C-S-H. Additionally, spectrum 4 show high concentration of Si and Al as in Figure 7b which represent unreacted fly ash.

The unreacted fly ash remains present between AAFA matrix as confirmed by the FESEM image in Figure 8a. The AAFA specimen’s elemental compositions cast in river water (Figure 8b) are represented by spectrum 27, 28, and 29. From the three different spots, the Ca and Si are predominant indicating the existing of calcium silicate hydrate (C-S-H). 

The microstructure of specimens cast in lake water (Figure 9a) showed micro-crack and it is believed to be due to sample preparation for FESEM. For spectrum 54, it is dominated by Ca, Na, and Si, which signifying the formation of C-S-H. However, spectrum 55 is mostly dominated by Ca, Si, Al, Fe and by referring to Figure 9b, the location of this spectrum is on spherical shape of fly ash. The elemental composition of spectrum 56 is predominated by Si, Al, and Fe.

In Section 3.4, C-S-H presence is confirmed via XRF analysis due to the increment in Ca and Si content. Additionally, the same finding also noted in EDS analysis where the C-S-H supported the compressive strength by acting as a micro-aggregate in the AAFA which produced denser AAFA matrix. The formation of C-S-H started from dissolution of Ca from the source material where some of the Ca will precipitate in the form of calcium hydroxide (Ca(OH)_2_) and C-S-H. Likewise, Si species also favourably to react with dissolved Ca rather than polymerise with soluble Al [58,59]. Hence, the presence of excessive Al will force out from Ca-rich area into the AAFA network. Past research also found that C-S-H gel contribute to the strength development at later age such as at 28th days [57,58].

Through EDS analysis, the existence of Fe was noticeable from all specimens and was reconfirmed by the XRF result. The high percentage of Fe in AAFA network due to involvement as substitution for Al, which leads to formation ferro-sialate-siloxo and ferro-sialate-disiloxo poly binders where Ca^2+^ and Na^+^ act as charge-balancing cations [57].

## 4. Conclusions

The use of concrete for underwater placement is a significant challenge due to washout effect as well as the presence of various ions in the water which can influence the properties of concrete. The present study analysed the strength, changes in water pH, and AAFA setting time when go through underwater placement in seawater and freshwater. The chemical composition of AAFA paste is analysed using XRF and EDS. The AAFA can be used as binder for underwater concrete without the addition of anti-washout admixture (AWA). The maximum compressive strength of 71.10 MPa was obtained from the specimens cast in seawater on 28th days. It demonstrates 12.11% of strength reduction compared to specimens cast in dry condition and according to JSCE standard, the AAFA specimens are qualified to use for underwater casting. It was found that the presence of Cl- ions in seawater leads to formation of calcium chloride (CaCl2) which acts as accelerator for early setting time and strength development.

## Figures and Tables

**Table 1:** Mix design for AAFA paste.

Parameter	Indicator
Ratio fly ash/alkaline activator	2
Ratio waterglass/NaOH	2.5
Mass of fly ash (wt.%)	66.7
Mass of NaOH solution (wt.%)	9.6
Mass of sodium silicate solution (wt.%)	23.8

**Table 2:** Setting time of AAFA cast underwater.

Types of Water	Setting Times (minutes)	
	Initial	Final
Dry Condition	31 ± 0.5	40 ± 0.5
Seawater	26 ± 0.5	35 ± 0.5
River Water	30 ± 0.5	37 ± 0.5
Lake Water	28 ± 0.5	35 ± 0.5

**Table 3:** Comparison of chemical composition for all specimens.

		AAFA Paste (wt. %)			
Composition	Fly Ash	Dry Condition	Seawater	River Water	Lake Water
SiO_2_	30.80	34.30	34.60	34.20	34.00
Al_2_O_3_	13.10	10.60	10.80	10.70	10.70
CaO	22.30	21.50	22.00	21.20	21.20
Fe_2_O_3_	22.99	24.75	24.38	23.64	23.47
MgO	4.00	3.10	3.10	3.00	3.20
TiO_2_	0.89	0.94	0.93	0.88	0.88
K_2_O	1.60	1.43	1.42	1.30	1.33
SO_3_	2.67	2.02	1.20	0.93	0.91
MnO	0.21	0.22	0.22	0.21	0.20
Si/Al ratio	2.35	3.24	3.20	3.20	3.18
Ca/Si ratio	0.72	0.63	0.64	0.62	0.62
Fe/Si ratio	0.75	0.72	0.70	0.69	0.69
Strength (MPa)	-	80.9	71.1	63.7	69.5
